# Mechanism of AI chatbot anthropomorphism on consumer impulse buying in live streaming scenarios based on SOR framework

**DOI:** 10.1371/journal.pone.0357787

**Published:** 2026-09-11

**Authors:** Jingqi Huang, Wasim Ahmad

**Affiliations:** 1 UCSI Graduate Business School, UCSI University, Malaysia; 2 ARUCAD Research Centre, Arkin University of Creative Arts and Design, Mersin, Northern Cyprus, Turkey; Pingdingshan University, CHINA

## Abstract

As artificial intelligence (AI) is integrated into real-time e-commerce, it is necessary to study how AI virtual hosts affect consumers’ immediate purchase decisions. This research is based on the Stimulus-Organism-Response (S-O-R) framework and explores the psychological mechanism by which the characteristics of AI hosts influence impulsive purchases. The scenario-based experiments show that AI anthropomorphism (ANT) and social conversation cues (SCC) affect impulsive purchases through two mediating states: cognitive trust and emotional arousal. The results indicate that the association between SCC and trust and arousal is stronger than that of visual anthropomorphism, and multiple group analyses show that this mechanism is roughly consistent across gender groups, but there are differences between the product participation and AI experience groups. This research expands upon previous work by testing visual and verbal anthropomorphic cues as parallel stimuli in live-streaming business, and provides guidance for the design of AI virtual hosts.

## 1. Introduction

**Fig 1:**
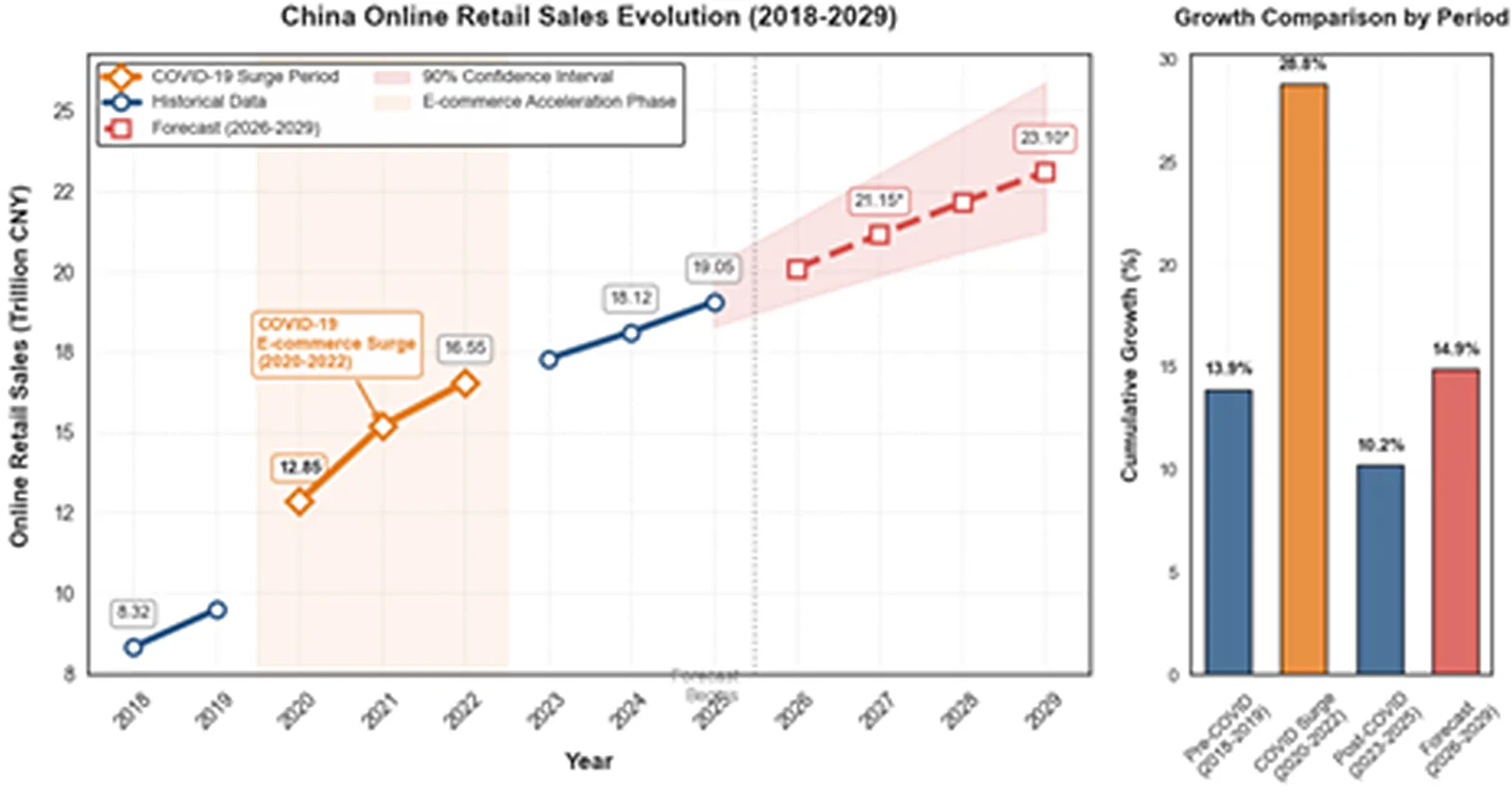
Analysis of Online Retail Sales in China.

In the macro evolution picture of the digital economy, live-streaming e-commerce has risen from a marginal marketing tactic to an infrastructure base that reconstructs the logic of commercial interaction [1], thus completely eliminating the temporal and spatial constraints of traditional retail [2]. According to the authoritative data calculation of iiMedia Research (see Fig 1), the scale of China's online retail market continues to expand and is expected to climb to a high of 19.05 trillion yuan in 2025. At present, this industry is in a critical window period of kinetic transformation from extensive “wild growth” to intensive “high-quality development.” Under the multi-party synergy of capital support, platform governance and policy regulation, a standardized and systematic industrial development framework is being accelerated to be established.

The current live-streaming e-commerce ecosystem is undergoing a structural paradigm reshaping from human-driven to “AI intermediation” [3,4]. AI agents have transcended the realm of instrumental rationality and have been restructured into interaction subjects with quasi-social attributes, dominating the key link from conversational interaction to business transformation in millisecond-level responses [5].

However, the way in which artificial personification shapes consumers’ psychology and triggers impulsive purchases still has not been fully explained. Existing research leaves three unresolved issues. Firstly, it has not fully clarified why personification design can increase social intimacy and also raises concerns about the horror valley effect [6]. Secondly, it usually regards personification as a single structure, which masks the different effects of visual presentation and language interaction [7,8]. Thirdly, it lacks a comprehensive explanation of how cognitive evaluation and emotional arousal work together in live consumption [9,10].

Given these gaps, this study applies the S-O-R framework to test the impulse purchase mechanism driven by artificial intelligence. Firstly, it distinguishes the visual and dialogue dimensions of the artificial intelligence host and tests their respective paths. Secondly, it examines the parallel mediating role of cognitive trust and emotional arousal. Thirdly, it links the research results with the actual design of artificial intelligence hosts in real-time streaming commerce, especially the coordination of appearance authenticity and conversation responsiveness.

## 2. Literature review

In the context of the era when artificial intelligence is reshaping service interaction, personification has transcended the single category of aesthetic design and risen to become a core variable in the ontological understanding of human-computer interaction [11]. Although the view based on the social response theory holds that highly anthropomorphic features can solidify the foundation of trust by enhancing the perception of warmth [12,13], its effectiveness does not increase linearly but rather presents a complex “double-edged sword” effect. Excessive personification may trigger the “uncanny valley effect” at the biological instinct level [14]. This paradox has prompted the academic community to start examining the heterogeneity of the interaction between visual representation and behavior, pointing out that “verbal personification” is often more effective in avoiding defensive psychology and optimizing marketing effectiveness than simple “visual simulation” [15,16].

From the perspective of the S-O-R theory, the mechanism by which artificial intelligence personification affects consumer behavior depends on the cognitive and emotional pathways. On the cognitive pathway, personified interaction builds trust by creating a sense of social presence and reducing service uncertainty [17,18]. On the emotional pathway, the artificial intelligence live streaming stream serves as an emotional cue in a high-engagement shopping environment, enhancing the flow experience and impulse purchase intention [19–22].

**Fig 2:**
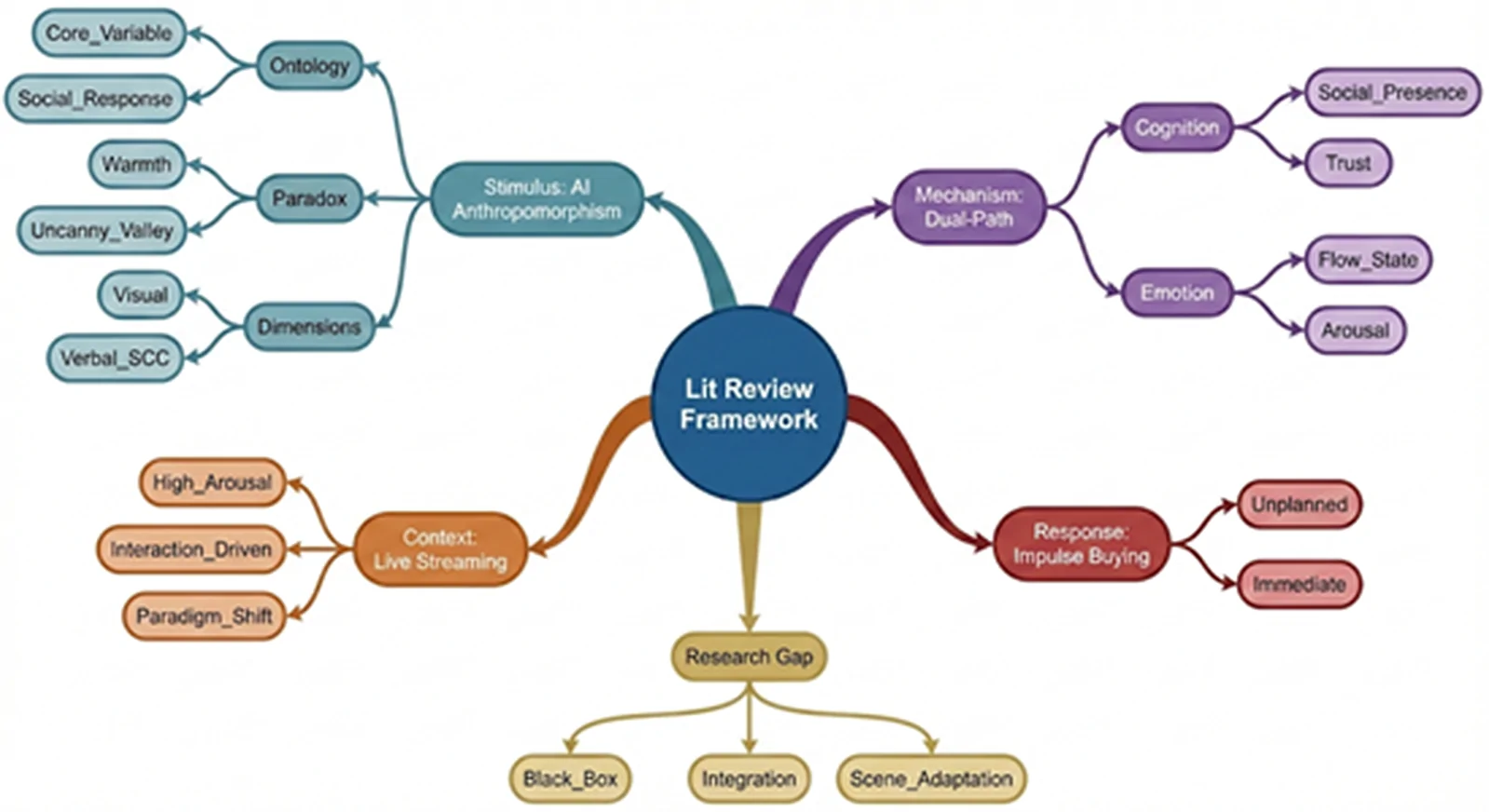
Architecture Diagram of the literature review.

In the context of live e-commerce, impulse purchases are largely influenced by the interaction [23]. Compared to static visual fidelity, the dialogue-level SCC can better predict relationship harmony and unplanned consumption [16]. Although human streaming still has an advantage in perceiving intimacy, virtual avatars are changing user expectations in specific business scenarios [24–26]. As shown in Fig 2, this study integrates visual and language-based personification cues within the S-O-R framework and examines the dual path from artificial intelligence stimulation to impulse purchase through trust and arousal.

## 3. Theoretical framework & Hypotheses

**Fig 3:**
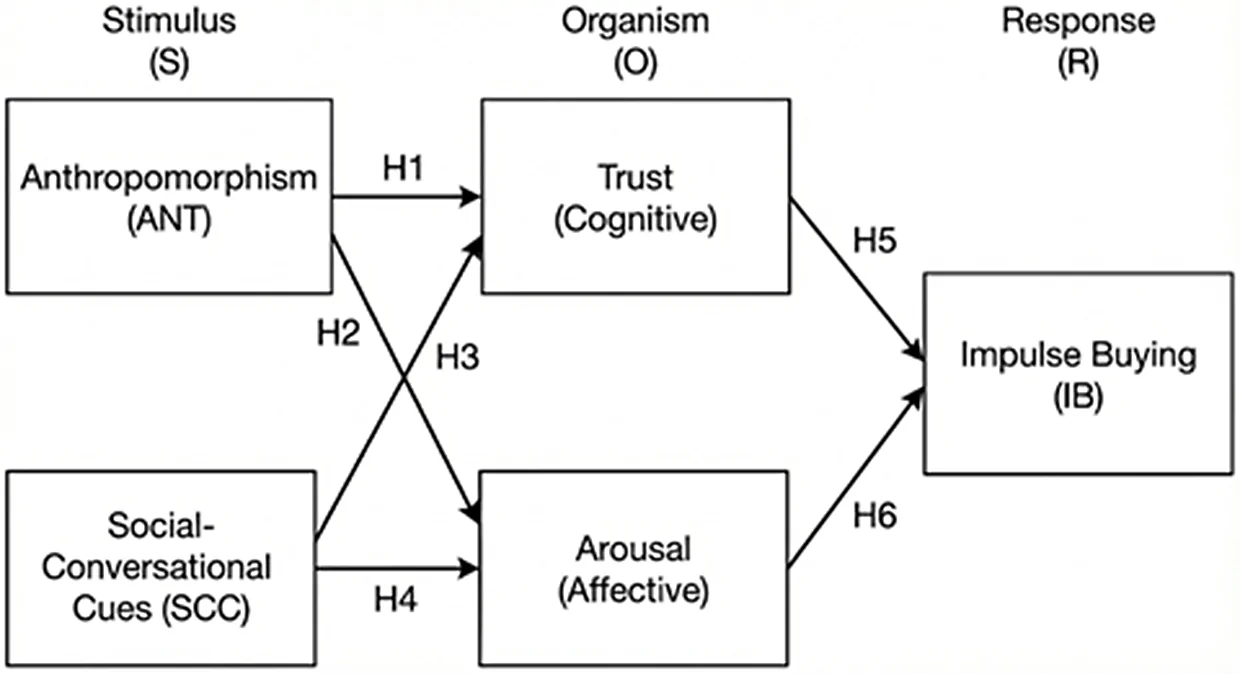
Research Analysis Framework.

This study adopts the S-O-R model from environmental psychology as its theoretical foundation [26]. The S-O-R framework can precisely capture how dynamic environmental cues drive consumers’ immediate decisions through psychological intermediary mechanisms. As shown in Fig 3, in the specific context of live-streaming e-commerce, we decompose the characteristics of the AI host into ANT and SCC as external stimuli; we decompose the consumers’ psychological responses into Trust at the cognitive level and Arousal at the emotional level as the body state; and the final Impulse Buying is the behavioral response.

### 3.1. ANT and Organismic Reactions

Personification, as the core dimension of AI technology's perception, refers to the extent to which users attribute human appearance, demeanor, and mental characteristics to non-human objects [27]. The personification attribute of text chat robots is a key predisposing factor for building consumer trust. When AI presents human-like appearance or role characteristics, it triggers users’ social heuristic processing, causing them to tend to transfer the trust mechanism for humans to the AI. Studies have shown that the perceived personification of virtual hosts significantly positively affects consumers’ trust levels, as the personified image reduces psychological distance [28]. Although personification may trigger the horror valley effect in certain situations, in service scenarios, appropriate personification design usually enhances users’ acceptance and trust. Therefore, we believe that in live streaming scenarios, the personified image of AI hosts helps establish a cognitive foundation for trust.

On the other hand, personification is also a strong source of emotional stimulation [29]. Highly personified AI services can significantly enhance the flow experience of online customers, which often accompanies a high level of emotional arousal. The research on virtual person live streaming has confirmed that the intention of quasi-social interaction with AI hosts and the “coolness” are highly correlated, and this novel technological experience can directly induce the awakening state of the audience [30]. The intelligence level of AI virtual idols can effectively reduce psychological distance, and this psychological closeness is easily transformed into an excited and agitated emotional reaction in visual-dominated live streaming. Therefore, personification not only affects rational trust but also directly affects emotional arousal.

Based on the above discussion, the following hypotheses are proposed:

H1: ANT positively affects Trust.

H2: ANT positively affects Arousal.

### 3.2. SCC and Organismic Reactions

SCC refers to the humor, empathy, reciprocity, self-disclosure and immediate response capabilities demonstrated by artificial intelligence in interaction. Compared with purely physical anthropomorphicization, similar human-like conversational tone can significantly predict users’ trust beliefs [31]. Social cues at the language level convey warmth and response. Artificial intelligence with human communication style is more likely to be regarded as having social capabilities, which helps reduce users’ doubts and support the formation of trust. In human-computer interaction, relationship norms regulate the influence of anthropomorphic attributes on trust, meaning that dialogue cues that conform to social expectations help maintain the trust relationship [32].

Dialogue cues also affect emotional responses. When the virtual host demonstrates behavioral authenticity through natural interaction, consumers’ purchase intentions may increase. This effect often accompanies emotional resonance. The perceived response affects the emotional response of the audience [33]. The immediate, humorous and empathetic responses of the artificial intelligence host can create a positive interaction atmosphere and increase the audience's excitement. Therefore, the emotional adaptability of artificial intelligence affects the user experience and psychological activation.

Based on the above discussion, the following hypotheses are proposed:

H3: SCC positively affects Trust.

H4: SCC positively affects Arousal.

### 3.3. The dual mediation mechanism: trust and arousal

Trust in this model represents a rational cognitive path. In the real-time streaming e-commerce environment, consumers face information overload, and trust is a heuristic method for simplifying decisions [34]. Trust beliefs link the attributes of chatbots with impulsive purchases. When consumers trust artificial intelligence recommendations, they reduce cognitive scrutiny and are more willing to follow purchase suggestions. Previous research has also shown that trust can predict impulsive purchase intentions in virtual artificial intelligence streaming [25]. Therefore, the trust formed through the anthropomorphic features of artificial intelligence can reduce perceived risks and reduce rational resistance to impulsive purchases.

Awakening represents the emotional path. According to environmental psychology, impulsive purchases are closely related to immediate emotional states [35]. High arousal weakens self-control and increases purchase impulsiveness. When consumers are emotionally close to the artificial intelligence host and experience excitement during interaction, they are more likely to form a purchase intention immediately. Therefore, trust and awakening constitute two organizational paths in the model.

Based on the above discussion, the following hypotheses are proposed:

H5: Trust positively affects Impulsive Buying.

H6: Arousal positively affects Impulsive Buying.

H7: Trust acts as a mediator between ANT/SCC and Impulsive Buying.

H8: Arousal acts as a mediator between ANT/SCC and Impulsive Buying.

## 4. Research design

### 4.1. Research Design

This study used a scenario-based experimental questionnaire. It followed a 2 (ANT: high vs. low) x 2 (SCC: high vs. low) between-subjects design, in which participants were randomly assigned to one of four simulated live-streaming conditions.

To construct an experimental situation with high ecological validity, we developed a set of simulated video scripts featuring AI hosts promoting products, based on the interface characteristics of current mainstream live streaming platforms such as TikTok. These scripts strictly controlled irrelevant variables (such as product type, price, and promotional intensity), with the only change being the anthropomorphic presentation and dialogue interaction strategy of the AI hosts. The specific parameter Settings of the video are shown in Table 1.

**Table 1:** Specification of Experimental Stimuli Parameters.

Dimension	Parameter	High ANT / High SCC Condition	Low ANT / Low SCC Condition
Visual Representation	Texture Mapping	Subsurface Scattering (SSS) Material	Standard Lambertian Material
	Facial Kinematics	48 Blendshapes	Static Mesh
	Body Language	High-frequency Natural Gestures	Minimal Mechanical Movements
Auditory Representation	TTS Model	Transformer-based Expressive TTS	Standard Parametric TTS
	Prosody	Emotional Prosody	Mechanical Prosody
Social Dialogue Cues	Pronoun Usage	Second-person Dominant	Third-person Dominant
	Emotional Word Density	> 15%	< 2%
	Response Strategy	Active Empathy	Passive Retrieval
Control Variables	Live Stream Background	Unified Warm-toned Home Environment	Consistent across conditions
	Product Type	Low-involvement Hedonic Product	Consistent across conditions
	Video Duration	Strictly controlled at 15s (±2s)	Consistent across conditions

Participants were required to fill out the questionnaire on the online survey platform immediately after watching the above simulated live streaming clips. To ensure the validity of the data, we set strict screening questions at the beginning of the questionnaire to exclude samples who had never encountered AI customer service, and at the end of the questionnaire, we set attention test questions to minimize the random answering behavior of the participants.

Using a pre-test involving 50 participants, we examined whether the stimulus materials could be understood, and whether the low SCC condition still conveyed the basic conversation rather than completely lacking social interaction. The low SCC condition retained the product explanation and simple response functions, but reduced the emotional language.

### 4.2. Sampling and Data Collection

The overall objective of this study was to target “digital natives” with rich digital living experiences and consumers with live-streaming shopping habits. Data collection relied on the authoritative online sample library platform Credamo, which has a large pool of high-quality participants and can precisely match user profiles that fit the characteristics of this study through algorithms.

Before the formal survey, we conducted a small-scale pre-test (N = 50) to test the semantic clarity of the questionnaire items and made corrections to some expressions based on the feedback.

The formal investigation adopted a combined strategy of snowball sampling and stratified random sampling. A total of 500 questionnaires were distributed through online channels. After excluding invalid questionnaires due to short response times, 418 valid samples were obtained. The demographic characteristics included gender, age, income level, and frequency of online shopping. The majority of the respondents were aged between 18 and 35, which reflects the core audience of current streaming e-commerce.

### 4.3. Measurement of Variables

This study draws on existing research designs for scales [16,21,26], and all the scale items strictly follow the standard back-translation procedure [36]. Bilingual scholars were invited to conduct Chinese-English translation and semantic comparison to ensure conceptual equivalence in cross-cultural contexts. All items are scored using the Likert 7-point scale, ranging from “1 = strongly disagree” to “7 = strongly agree.”

Personification (ANT): As the core independent variable, it mainly measures the degree to which consumers perceive AI hosts to possess human characteristics. It includes 4 items, focusing on the degree of anthropomorphism of AI hosts in appearance, actions, and expressions.

Social-Conversational Cues (SCC): It measures the perceived social attributes of AI hosts at the language interaction level. Four items cover conversational warmth, friendly address, reciprocity of responses, and empathy expression.

Trust (TRU): As the mediating variable of the cognitive path, it mainly assesses consumers’ perception of the ability and integrity of AI hosts. It includes 3 core items, measuring from the dimensions of ability, integrity, and kindness.

Awakening (ARO): As the mediating variable of the emotional path, it aims to capture the emotional activation state of consumers during the interaction. It includes 3 items, mainly describing the degree to which the subjects feel excited, stimulated, and emotionally elevated.

Impulse Buying (IB): As the dependent variable, it measures the immediate purchasing impulse of consumers in the live streaming context. It includes 4 items, covering both the desire to purchase and the intention to purchase.

Control Variables (CV): To eliminate the confounding effects of exogenous variables, this study incorporates gender, age, average monthly online shopping expenditure, frequency of live streaming shopping, AI usage experience, product involvement, and perception of promotion intensity into the control variable system.

The definitions of the main variables are shown in Table 2.

**Table 2:** Measurement of Variables.

Variable Type	Variable Name	Abbr.	Source Reference
Independent Variable	AI Anthropomorphism	ANT	Zhou & Li (2025) [26]
	Social Dialogue Cues	SCC	Ooi et al. (2025) [16]
Mediating Variable	Cognitive Trust	TRU	Zhou & Li (2025) [26]
	Emotional Arousal	ARO	Zhang et al. (2024) [21]
Dependent Variable	Impulse Buying Intention	IB	Ooi et al. (2025) [16];Zhou & Li (2025) [26]
Control Variable	Gender	CVs	Standard Items
	Age		
	Aaverage Monthly Online Shopping Expenditure		
	Frequency of Live Streaming Shopping		
	AI Usage Experience		
	Product Involvement		
	Perception of Promotion Intensity		

### 4.4. Model Construction

This study used Model 1 to verify the direct effect, as shown in Equation (1):


$$\begin{array}{c} DV=\beta_{\text{1}}IV+\sum_{j=\text{1}}^{n}\gamma_{\text{1}j}CV_{j}+\varepsilon_{\text{1}} \end{array}$$ (1)

Here,$\varepsilon_{\text{1}}$ represents the residual. The following model is the same.

Verify the Intermediate Effect using in Equations (2)-(3):


$$\begin{array}{c} \text{Med}=\beta_{\text{2}}\text{IV}+\sum_{\text{j}=\text{1}}^{\text{n}}\gamma_{\text{2j}}\text{C}{\text{V}}_{\text{j}}+\epsilon_{\text{2}} \end{array}$$ (2)

$$\begin{array}{c} \text{DV}=\beta_{\text{3}}\text{IV}+\text{Med}+\sum_{\text{j}=\text{1}}^{\text{n}}\gamma_{\text{3j}}\text{C}{\text{V}}_{\text{j}}+\epsilon_{\text{3}} \end{array}$$ (3)

Given that the complexity of consumer behavior is often masked by the average effect of the sample population, this study introduces multi-group analysis to reveal the boundaries of parametric heterogeneity among different trait groups.

We divided the total sample into g subgroups based on specific moderating variables and constructed a non-parametric Henseler's MGA test statistic. This method generates an empirical distribution through the Bootstrap resampling technique to test whether there are significant differences in the path coefficients between $\beta^{\left(g\text{1}\right)}$ and $\beta^{\left(g\text{2}\right)}$ in different groups, as shown in Equation (4):


$$\begin{array}{c} P\left(\beta^{\left(g\text{1}\right)}>\beta^{\left(g\text{2}\right)}\right)=\text{1}-\frac{\text{1}}{B}\sum_{b=\text{1}}^{B}I\left(\beta_{b}^{(g\text{1})*}>\beta_{b}^{(g\text{2})*}\right) \end{array}$$ (4)

If the test result P < 0.05 or P > 0.95, it indicates that there are significant structural differences in the AI anthropomorphic mechanism among different groups.

The design based on random scenarios provides the main basis for causal explanations. The Logit model is used to estimate the probability of exposure to high-intensity artificial intelligence stimulation conditions based on the observed demographic and behavioral covariates, as shown in Equation (5):


$$\begin{array}{c} P\left(X_{i}\right)=Pr\left(Treatment_{i}=\text{1}|CVs_{i}\right)=\frac{exp\left(\alpha +\gamma CVs_{i}\right)}{\text{1}+exp\left(\alpha +\gamma CVs_{i}\right)} \end{array}$$ (5)

Monte Carlo simulation is used to evaluate the stability of the intermediate estimation under repeated sampling. This simulation employed 20,000 iterations and the covariance matrix of the benchmark model. RMSE and MAD were used to assess the numerical stability, as shown in Equation (6):


$$\begin{array}{c} RMSE\left(\hat{\beta }\right)=\sqrt{\frac{\text{1}}{K}\sum_{k=\text{1}}^{K}{\left(\hat{\beta_{k}}-\beta_{true}\right)}^{\text{2}}} \end{array}$$ (6)

### 4.5. Analytical Strategy

This study employed Partial Least Squares Structural Equation Modeling (PLS-SEM) as the main analytical tool [37], and selected SmartPLS 4.0 [38] as the software. The measurement model evaluation utilized factor loadings, Cronbach's alpha, composite reliability (CR), and average variance extracted (AVE) to test convergent validity and discriminant validity. The structural model evaluation utilized path coefficients, determination coefficients, and predictive correlations to validate the hypotheses, and employed guided resampling to test the significance of the mediating role of trust and arousal.

## 5. Results

### 5.1. Reliability and validity test

As shown in Table 3, the Cronbach's α coefficients and CR of all latent variables are significantly higher than the threshold of 0.70, and the AVE of all items have exceeded the critical point of 0.50. This indicates that the scale has extremely high internal consistency.

**Table 3:** Reliability and convergent validity.

Latent Constructs	Items	Factor Loading	Cronbach’s α	CR	AVE
ANT	ANT1	0.842	0.887	0.921	0.745
	ANT2	0.865			
	ANT3	0.891			
	ANT4	0.853			
SCC	SCC1	0.876	0.895	0.927	0.761
	SCC2	0.889			
	SCC3	0.834			
	SCC4	0.890			
TRU	TRU1	0.855	0.863	0.916	0.784
	TRU2	0.901			
	TRU4	0.898			
ARO	ARO1	0.823	0.841	0.904	0.758
	ARO2	0.884			
	ARO3	0.902			
IB	IB1	0.867	0.912	0.938	0.791
	IB2	0.895			
	IB3	0.881			
	IB4	0.913			

More importantly, as shown in Table 4, the HTMT values between all the constructs are all below the conservative threshold of 0.85.

**Table 4:** Discriminant Validity.

Constructs	ANT	SCC	TRU	ARO	IB
ANT	–				
SCC	0.612	–			
TRU	0.543	0.687	–		
ARO	0.489	0.556	0.412	–	
IB	0.521	0.601	0.578	0.634	–

**Fig 4:**
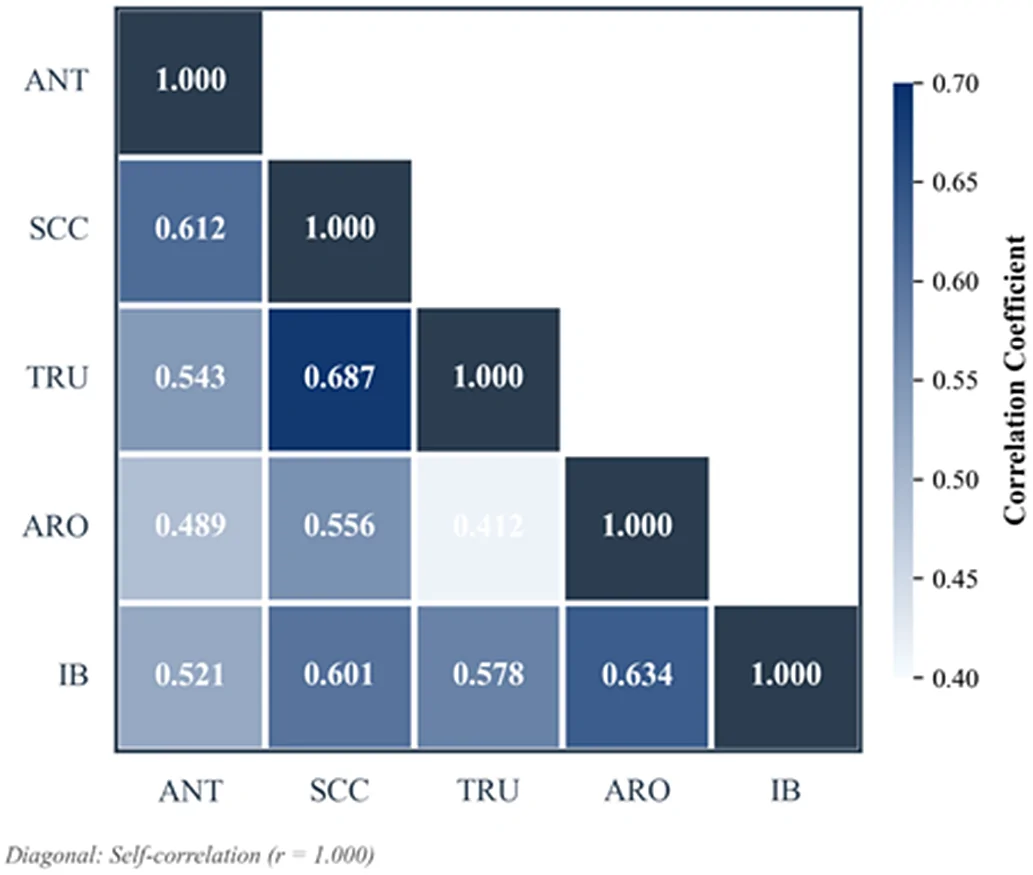
HTMT Values Heat Map.

As shown in Fig 4, the HTMT results indicate that anthropomorphism, social-conversational cues, trust, and arousal are statistically distinct constructs, and that the model does not show a serious multicollinearity problem.

### 5.2. Structural model estimation

After confirming the validity of the measurement model, self-evaluation was used to test the structural paths. As shown in Table 5, trust and arousal jointly explained approximately 58.4% of the variance in impulsive purchasing, indicating that the S-O-R model has sufficient predictive ability in this situation. The coefficients of SCC in terms of trust and arousal are stronger than those of ANT, suggesting that conversational responsiveness and social tone have a stronger association with consumer responses than the individual aspects of appearance realism. Both trust and arousal significantly affect impulsive purchasing, with the coefficient of arousal being slightly higher. This pattern is consistent with the high arousal nature of live-streaming business.

**Table 5:** Path Coefficients and Hypothesis Testing.

Hypothesis	Path Relationship	Std. Beta (β)	T-Value	P-Values	Decision
H1	ANT → TRU	0.245	4.123	0.000***	Supported
H2	ANT → ARO	0.218	3.567	0.000***	Supported
H3	SCC → TRU	0.482	8.912	0.000***	Supported
H4	SCC →ARO	0.395	6.458	0.000***	Supported
H5	TRU → IB	0.356	5.876	0.000***	Supported
H6	ARO → IB	0.412	7.021	0.000***	Supported
Direct effect	ANT→IB (controlling TRU and ARO)	0.064	1.182	0.237	Not supported
Direct effect	SCC→IB (controlling TRU and ARO)	0.091	1.611	0.107	Not supported

### 5.3. Intermediary testing

The mediation analysis followed the guided framework proposed by Preacher and Hayes, rather than relying solely on the causal steps method of Baron and Kenny [39–41]. As shown in Table 6, the confidence intervals of all four indirect paths did not include zero. After including trust and arousal in the model, the direct effects of ANT and SCC on impulsive buying were no longer significant, as shown in the additional rows of Table 5. These results support a complete mediation model, in which the organizational state is the proximal driver of impulsive buying in the S-O-R framework.

**Table 6:** Specific Indirect Effects.

Mediation Path	Indirect Effect	Result	95% CI	T-Value
H7a	ANT → TRU → IB	0.087	[0.045, 0.138]	3.214
H7b	SCC → TRU → IB	0.171	[0.112, 0.235]	5.102
H8a	ANT→ ARO → IB	0.090	[0.042, 0.145]	3.015
H8b	SCC → ARO → IB	0.163	[0.108, 0.221]	4.897

**Fig 5:**
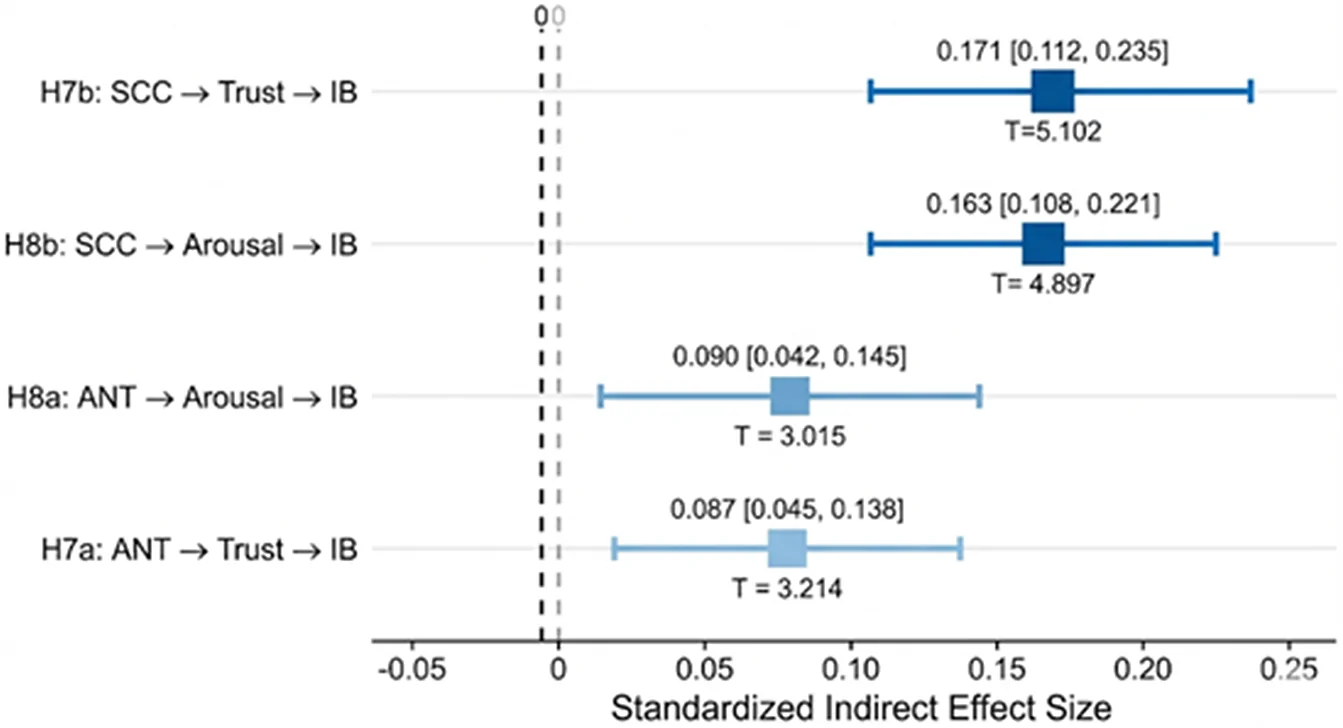
Mediating Effect Forest Plot.

Furthermore, Fig 5 shows two mediation paths. In the cognitive path, consumers reduce perceived risk when they regard the AI host as human-like and conversationally competent. In the emotional path, social and empathetic interaction increases arousal during the live-streaming experience. The larger indirect effects associated with SCC indicate that conversational cues are especially relevant for impulse buying in short, high-interaction live-streaming contexts.

### 5.4. Multi group testing

After verifying the mediating mechanism, this paper uses gender as the grouping variable for multi-group analysis. As shown in Table 7, the core paths do not differ significantly between male and female participants. This result indicates that the proposed psychological mechanism is stable across gender groups in this sample.

**Table 7:** Multi-Group Analysis Results(gender).

Structural Path	Male(β_M)	Female(β_F)	P-Value	Result
ANT → TRU	0.231	0.258	0.412	Not Sig.
SCC → TRU	0.495	0.468	0.389	Not Sig.
ANT → ARO	0.205	0.229	0.456	Not Sig.
SCC → ARO	0.410	0.382	0.321	Not Sig.
TRU → IB	0.368	0.342	0.487	Not Sig.
ARO → IB	0.398	0.425	0.354	Not Sig.

Based on the refined likelihood model, the samples were divided into a high participation group (N = 226) and a low participation group (N = 192) according to their average values. As shown in Table 8, consumers in the high participation scenario relied more on the trust mechanism, and the artificial intelligence anthropomorphic features played the role of a capability signal. In the low participation scenario, ANT and SCC were more used as peripheral cues, enhancing arousal and impulsive purchasing.

**Table 8:** Multi-Group Analysis Results(Product Involvement).

Structural Path	High Inv. (β_H)	Low Inv. (β_L)	P-Value	Result
ANT → TRU	0.445	0.189	0.002***	High > Low
SCC → TRU	0.392	0.205	0.018*	High > Low
ANT → ARO	0.210	0.478	0.005**	Low > High
SCC → ARO	0.315	0.502	0.024*	Low > High
TRU → IB	0.522	0.214	0.000***	High > Low
ARO → IB	0.245	0.565	0.000***	Low > High

Based on the theory of technology acceptance and learning curve, the samples were divided into the artificial intelligence novice group (N = 245) and the artificial intelligence experienced user group (N = 173) to examine the changes in novelty effects. As shown in Table 9, high ANT had a stronger influence on the trust and arousal of AI novices. Among the experienced users, the effect of visual anthropomorphism weakened, while SCC had a stronger association with trust. This result indicates that as users gain experience with artificial intelligence, the focus of artificial intelligence host design may shift from realistic appearance to interaction quality.

**Table 9:** Multi-Group Analysis Results(AI Usage Experience).

Structural Path	Novice (β_N)	Expert (β_E)	P-Value	Result
ANT → TRU	0.389	0.224	0.042*	Novice > Expert
SCC → TRU	0.215	0.468	0.004**	Expert > Novice
ANT → ARO	0.455	0.188	0.001***	Novice > Expert
SCC → ARO	0.412	0.395	0.785	Not Sig.
TRU → IB	0.356	0.382	0.654	Not Sig.
ARO → IB	0.405	0.428	0.712	Not Sig.

**Fig 6:**
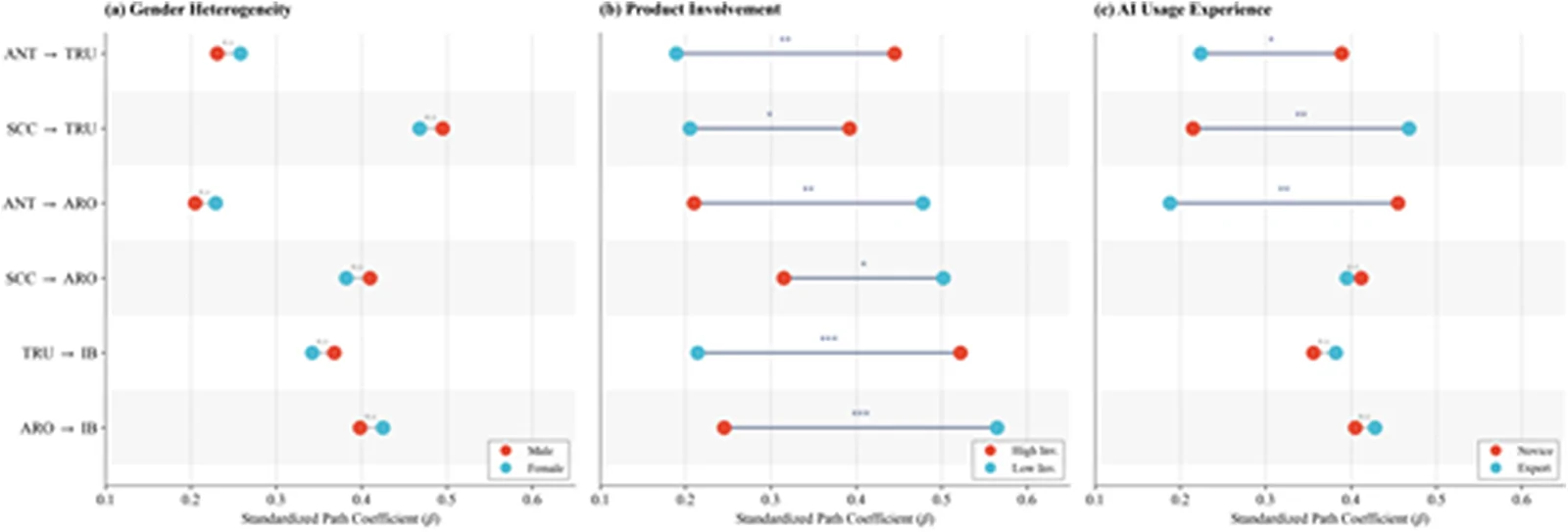
Group Dumbbell Diagram.

As shown in Fig 6, grouped dumbbell plots present the MGA results and show how the path coefficients vary across the moderating variables.

### 5.5. Robustness Test

**Fig 7:**
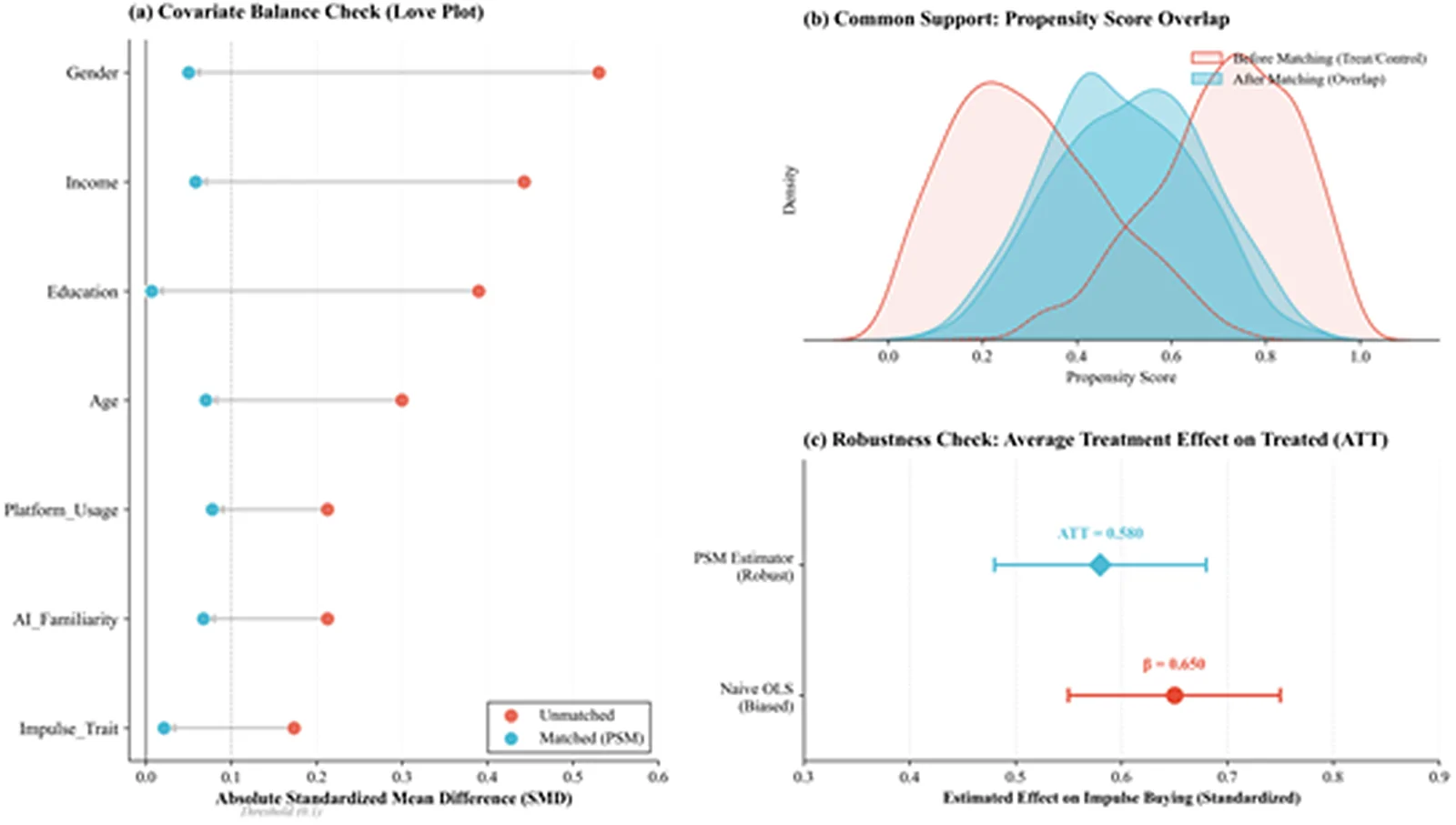
PSM Matching Results.

As a supplementary sensitivity check, PSM was used to examine whether the main outcome remained stable after balancing the observable covariates. As shown in Fig 7, the SMD of all covariates decreased below 0.1 after matching, and the distribution of propensity scores showed sufficient overlap. Therefore, the PSM results supported the stability of the experimental results.

**Fig 8:**
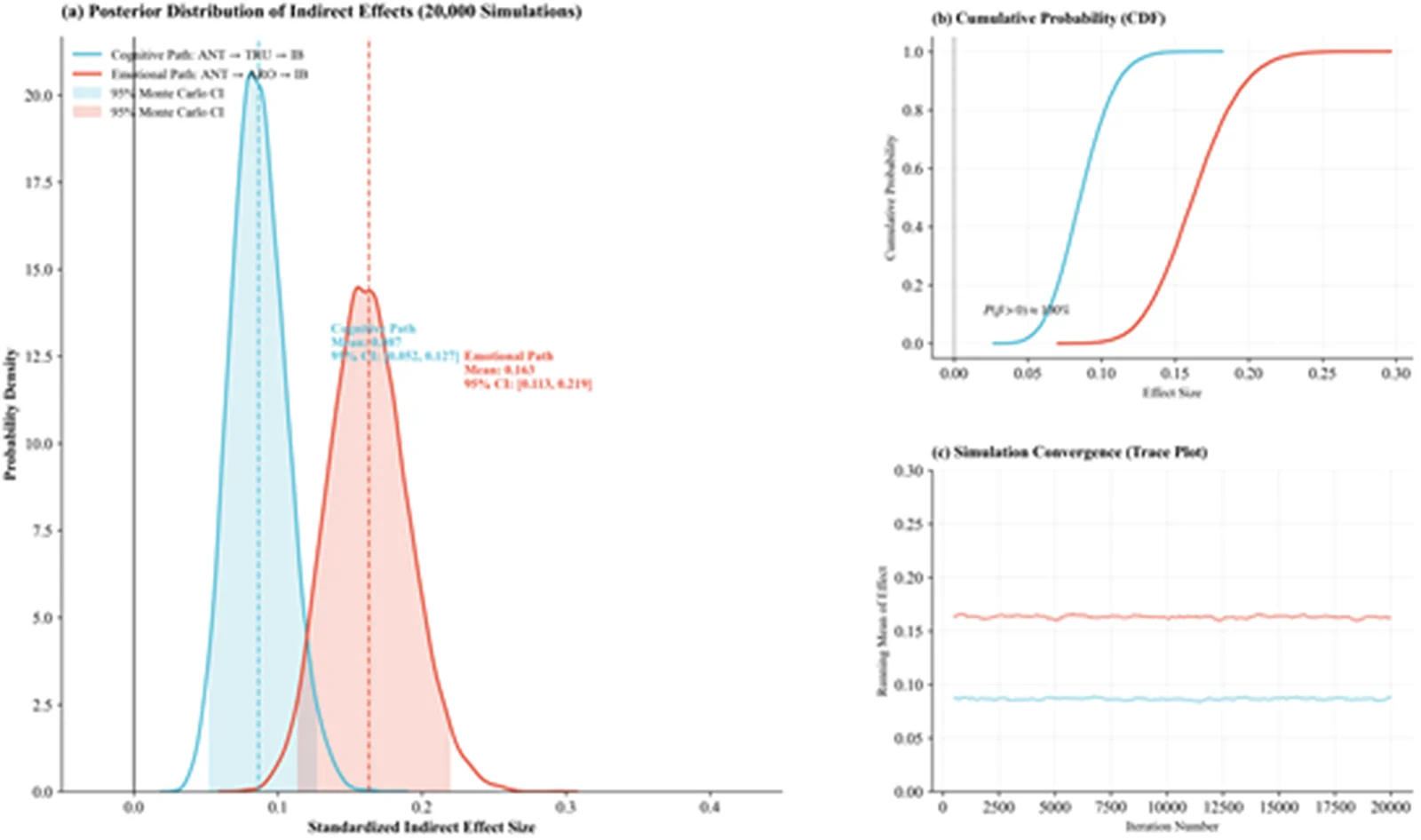
Monte Carlo Simulation.

Monte Carlo simulation was used to assess the stability of the mediation estimates. As shown in Fig 8, the simulated distributions of the two core mediating paths were above zero, and the 95% confidence intervals excluded zero. The result is consistent with the bootstrap mediation test and supports the stability of the dual-mediation results.

Since all the indicators were collected simultaneously from the same respondent, the issue of methodological differences remains relevant. Harman's single-factor test indicated that the first factor accounted for 28.4% of the total variance, which was lower than the traditional 50% benchmark. However, the diagnostic ability of this test was limited [42]. The VIF values ranged from 1.214 to 2.456, indicating that multicollinearity was not severe, but VIF could not rule out the common method variance. The experimental procedures and anonymous protocols reduced some of these concerns, and future research should include labeled variables or time-lagged measurements.

The original dual-mediation model was also compared with the competing model that removed the mediating variables. The original model showed higher explanatory power and better fit indices, supporting the use of trust and arousal as two organizational states within the S-O-R framework.

## 6. Conclusion and implications

Based on the S-O-R framework, this study examines how artificial intelligence virtual hosts in live e-commerce affect consumers’ impulsive purchases. The results show that cognitive trust and emotional arousal are parallel mediating paths, and the association between SCC and these two mediators is stronger than that of visual ANTs.

This study extends previous work and tests visual and verbal anthropomorphic cues as parallel stimuli in the context of live impulsive purchases. Early research discussed anthropomorphism in artificial intelligence services and chatbots, while this study places ANTs and SCC in the same empirical model and compares their paths through trust and arousal. The research results also indicate that the effect of visual anthropomorphism varies among the artificial intelligence experience group, suggesting an adaptation pattern as consumers become more familiar with artificial intelligence hosts.

These findings also have implications for platform operators. Investments in highly realistic visual modeling should be combined with conversational design. Artificial intelligence hosts need to have responsive, empathetic, and context-aware conversations to support trust and arousal in live interactions. Platforms can also adjust interaction strategies based on product engagement and users’ previous artificial intelligence experiences.

This study has several limitations. Firstly, although the experiments support a causal interpretation, the data was collected at a single point in time; future research can use longitudinal tracking and clickstream data to test whether novelty effects decline over time. Secondly, the sample is concentrated on Chinese consumers aged 18–35, so the research results should be tested in other regions, platforms, and age groups. Thirdly, the SCC conditions with low SCC show a large contrast in emotional word density and response strategies; future research should replicate this study at a more subtle manipulation level to test whether SCC operates as a continuous dimension. Fourthly, self-reported measures may still be affected by common method differences and social expectations. Future research should include coded variables, behavioral purchase data, or time lag indicators. Finally, the positive ANTs effect observed here may reflect the use of low engagement hedonic products and short video exposure, which may reduce the possibility of the revolting valley reaction. Longer interaction settings and more engaged products may produce different results.

## Supporting information

S1 Data Live streaming e-commerce questionnaire survey dataset. (XLSX)
